# Existing Smog in Lahore, Pakistan: An Alarming Public Health Concern

**DOI:** 10.7759/cureus.2111

**Published:** 2018-01-25

**Authors:** Ramsha Riaz, Khizar Hamid

**Affiliations:** 1 Dow Medical College, Dow University of Health Sciences (DUHS), Karachi, Pakistan

**Keywords:** smog, lahore, pakistan, air pollution, air quality, public health

## Abstract

Lahore, the second-largest and most polluted city in Pakistan, has been plagued by a heavy blanket of smog recently. The ever-growing urbanization and industrialization have contributed to the worsening air quality of the city. Smog, being hazardous to health, is leading to a rapid sprout in multiple health-related problems, as well as raising concerns about the long-term deleterious effects on public health. The current situation is expected to worsen due to the lack of an active action plan from the government's side and a failure of concerned authorities to take note of the urgency of the situation. Hence, we aim to highlight this pressing issue in the light of previously published articles, to alert the relevant authorities regarding the detrimental consequences smog can have on public health and urge them to take immediate action to avoid further damage.

## Editorial

Pakistan is the most urbanized country in South Asia [1], and its second-largest city Lahore, growing at a rate of 4% annually [2], is regarded as the most polluted city in Pakistan. Urban settlements are frequently plagued by smog in Asia, and Lahore is no exception. Following the pattern of last year, Lahore has once again been engulfed by a disturbingly heavy blanket of smog, shrouding the entire city and taking a toll on people’s lives. The exorbitant rise in automobiles, unchecked deforestation, expeditious urbanization, and unabated growth of industries [1-2] have contributed to this alarming situation over the years.

Smog accounts for a rapid sprout in fatal health problems, including exacerbation of asthma, allergies, eye infections, respiratory tract infections, and cardiac pathologies leading to premature death. Sughis et al. reported a concerning finding related to this situation, observing significantly higher levels of systolic and diastolic blood pressure in the school children of Lahore, exposed to high levels of air pollution [3]. This worrisome observation helps highlight the long-term deleterious effects on the health of the public.

The Pakistan Environmental Protection Agency (Pak-EPA) and the provincial EPAs are in charge of monitoring air pollution in Pakistan. In 2010, the Pak-EPA drafted the National Air Quality Standard (NEQS) for ambient air quality [2]. However, the proposed annual mean levels for the ambient particulate matter, PM_2.5_ and PM_10_, were higher than the stricter World Health Organization (WHO) guidelines, which are 10 μg/m^3^ and 20 μg/m^3^ respectively [2]. According to data, the levels of the ambient particulate matter reported in Lahore far exceed the recommended values of both WHO guidelines and NEQS guidelines [2,4]. A study conducted in Lahore over a period of 5 years, aiming to compare the level of fine particles with the aforementioned guidelines, concluded that the annual average PM_2.5_ of the areas studied was 136.5 ± 34.1 μg/m^3^ [4], which is roughly 14 folds higher than the WHO guidelines. This study also mentions that this level of particulate matter was comparable to one of the most polluted megacities of the world, Delhi, at 143.0 ± 17.8 μg/m^3^ [4]. This elucidates the worsening state of air pollution in the city of Lahore.

Furthermore, the fact that only around 1% of the country’s industrial establishments report their emissions [2] raises distressing concerns over the neglected air quality of the city and its effect on public health. Children are predominantly susceptible to these detrimental consequences. A study reporting the long-term effects of the great London Smog of 1952 concluded that exposure to smog during the first year of life increased the risk of childhood asthma by 19·87% [5].

Given the damage that smog can incur, it is imperative that prudent measures be undertaken to improve air quality. Most environmental regulatory organizations fall behind due to the lack of specialized equipment, standardized protocols, trained personnel, and funds [2]. The government could start by allocating appropriate funds for monitoring and reducing harmful emissions, carrying out nationwide afforestation programs, and switching to renewable resources. Considering the bleak outlook that the current situation portrays, the need of the hour is establishing a stringent action plan to prevent adverse outcomes on public health and reduce the economic burden on the health sector of the country.

Last but not the least, the public needs to be made aware of the possible health issues that can be encountered during this environmental hazard and educated on ways they can protect themselves and prevent exacerbations of pre-existing medical conditions. Public service messages on television, radio, and the Internet, along with the distribution of educational pamphlets and brochures can be a few of the effective steps for ensuring this. 
